# Clinical outcomes of triclosan-coated barbed suture in open hernia repair: a retrospective cohort study

**DOI:** 10.1007/s10029-024-03012-0

**Published:** 2024-04-12

**Authors:** F. Berrevoet, L. van Cauteren, N. Gunja, W. A. Danker, N.-D. Schmitz, J. Tomaszewski, L. Stern, A. Chandak

**Affiliations:** 1 University Hospital Gent, Corneel Heymanslaan 10, 9000 Ghent, Belgium; 2 Ethicon Inc., 1000 US-202, Raritan, NJ 08869 USA; 3 Certara, 100 Overlook Center, Suite 101, Princeton, NJ 08540 USA

**Keywords:** Stratafix, Triclosan-coated suture, Hernioplasty, Resource utilization, Surgical site infections

## Abstract

**Purpose:**

We assessed clinical outcomes of patients undergoing open hernia repair using STRATAFIX™ Symmetric, a barbed triclosan-coated suture (TCS; Ethicon), versus conventional polydioxanone suture (PDS) for abdominal wall closure.

**Methods:**

This single-center retrospective cohort study identified patients undergoing hernia repair. The site used PDS from 2013 to 2016 and switched exclusively to barbed TCS in 2017. Outcomes were assessed at 30, 60, and 90 days. Multivariate regression analyses and Cox proportional hazards models were used.

**Results:**

Of 821 hernia repairs, 446 used barbed TCS and 375 used conventional PDS. Surgical site infections (SSIs) were significantly less frequent with barbed TCS (60 days, 5.9% vs. 11.4%; *P* = 0.0083; 90 days, 5.9% vs. 11.7%; *P* = 0.006) and this remained consistent after multivariate adjustment (60 days, OR [95% CI]: 0.5 [0.3–0.9]; 90 days, 0.5 [0.3–0.9]). Among patients with SSI, deep SSIs were less frequent with barbed TCS (60 days, 9.1% vs. 35.7%; *P* = 0.022; 90 days, 9.1% vs. 34.9%; *P* = 0.0252). Barbed TCS significantly reduced the risk of perioperative complications (HR [95% CI]: 0.5[0.3–0.8]; *P* = 0.0058). Hospital length of stay was 2.5 days shorter with barbed TCS (mean [95% CI]: 5.7[4.9–6.6] vs. 8.2[7.3–9.1] days; *P* < 0.0001). No differences in reoperation rate over time were observed by type of suture (HR[95% CI]:1.3 [0.5–3.4]; *P* = 0.4793).

**Conclusions:**

This study showed that patients who underwent open hernia repair appeared to recover equally well regardless of the suture type. In addition, the use of barbed TCS was associated with significantly reduced risk of perioperative complications and hospital length of stay.

**Supplementary Information:**

The online version contains supplementary material available at 10.1007/s10029-024-03012-0.

## Introduction

The surgical approach for abdominal wall closure has the potential to prevent complications and reduce resource utilization associated with healthcare services [1]. Currently, there is a lack of consensus regarding the best suturing technique and material to use in open abdominal wall closure to prevent postoperative complications [2]. Surgeons are faced with a vast choice of materials due to the rapidly growing market for medical devices [3]. As shown by a recent network meta-analysis of 31 trials, while no suture material could be identified as optimal, the choice of suture has an impact on postoperative outcomes [4]. STRATAFIX™ Symmetric PDS Plus Knotless Tissue Control Device is a triclosan-coated barbed suture (barbed TCS) that provides strong, secure closure appropriate for high-tension areas, such as fascia [5, 6]. It can be used for abdominal midline closures in various procedures. In comparison to the polydioxanone (PDS) conventional suture, the barbed feature offers handling benefits to the surgeon by eliminating the need of knot tying and is also thought to improve clinical benefits and resource utilization [7, 8]. The goal of this study was to compare the clinical outcomes and resource utilization of patients undergoing open hernia repair using barbed triclosan-coated suture (TCS) or conventional PDS suture for abdominal midline closure.

## Methods

A non-interventional, retrospective, observational cohort chart review/data extraction study was conducted in patients undergoing open hernia repairs at the Gent University Hospital, Belgium, from January 2013 to August 2020. Conventional PDS suture (non-PLUS product used initially in the study period, with a switch to triclosan-coated PLUS product later) was used from January 1, 2013 to July 5, 2016 and was replaced by barbed TCS (STRATAFIX™ Symmetric suture; Ethicon) from July 6, 2017 to August 31, 2020.

All patients (18 years or older) with open hernias were included, regardless of hernia type (primary or incisional) with midline incision. The procedure in this series started with a midline incision that was carried down through the subcutaneous tissues until the anterior fascia is reached. The hernia sac was then identified and the fascia superior or inferior to the defect was entered. This fascial incision was lengthened to ensure that there was adequate exposure of the hernia defect. After careful dissection the peritoneum was always opened carefully with full exploration and adhesiolysis of the abdomen.

After the intraperitoneal portion of the procedure was completed, the retromuscular plane could be created for mesh placement. Observing the posterior rectus sheath from underneath, this layer was incised and dissected away from the rectus abdominis. Caution and use of blunt dissection during this portion of the case ensured preservation of the segmental neurovascular bundles and inferior epigastric vessels. If necessary, the fatty triangle behind the xiphoid process was opened and freed over 4–5 cm to increase later mesh overlap. If the hernia defect extended below the arcuate line, the transversalis fascia with peritoneum was dissected away from the anterior structures and carried down even more caudally into the spaces of Retzius and Bogros, exposing the pubic symphysis.

Laterally, the dissection typically extended to the lateral border of the rectus sheath in a typical repair according to Rives and Stoppa, but if necessary to extend mesh overlap a transverse abdominis release (TAR) was carried out on one or both sides across the linea semilunaris.

When full mobilization was achieved posteriorly, the posterior layer was then closed using a resorbable Vicryl 2/0 suture, and mesh (large pore monofilament polypropylene or polyvinylidene fluoride (PVDF)) was placed to reinforce the repair. Onlay was used in only a few patients in both cohorts, especially in the earlier time period when no TAR was yet performed. Open IPOM with anterior fascial closure was preferred over onlay mesh because of higher morbidity after anterior component separation technique. In exceptional cases, onlay mesh was used after closure of the anterior fascia when IPOM was rather contra-indicated (i.e., in Crohn’s disease patients). Once the mesh was in place with an overlap of at least 5 cm to all sides, it was fixed to bony edges using a multifilament suture (Ti-cron 1) or to the posterior layer using separate resorbable sutures (Vicryl 3/0) for each quadrant. Two closed suction drains were positioned above the mesh through lateral stab wounds and removed at postoperative day 2.

If there was persistent tension on the midline after retromuscular placement of mesh, an anterior component separation according to Ramirez was performed on one or both sides, but only when a TAR was not performed at that same side earlier. This maneuver allowed for added laxity of the midline, leading to less strain on the fascia when brought back together. Next, the anterior fascia was sutured together using PDS 1 or a barbed triclosan-coated suture 1 or 0 (Stratafix Symmetric PDS Plus 1 or 0), and the midline was completely reconstructed using mesh augmentation.

In case a bilateral anterior component separation technique was needed, in several cases an intraperitoneal mesh was placed to achieve wide mesh overlap covering both semilunar lines until the mesh reached the psoas muscle. In these cases, a running PDS 2/0 was used all around to fix the mesh to the peritoneum. In all cases, one or two suction drains were placed subcutaneously to prevent seroma formation and these were removed when daily output was less than 30 cc/day. An abdominal binder was used in all patients for 3 weeks to increase mobilization and patient comfort during activities.

Baseline characteristics (e.g., 1 year of demographic/comorbidity data and 5 years of abdominal surgery or midline laparotomy history) as well as clinical outcomes and resource utilization for the fixed follow-up durations of 30, 60, and 90 days were documented by the study site. Patients enrolled in clinical trials in the 1-year period prior to the date of the open hernia repair (index date) and patients undergoing emergency procedures were excluded.

Formal power calculations were not conducted as the study was not designed to detect differences in outcomes. Rather, the study sample size was based on the total number of patients that underwent an open hernia repair procedure at the center during the study period. Clinical outcome data were collected retrospectively on perioperative complications (wound dehiscence, surgical site infections [SSIs]), hernia recurrence, blood transfusion, and vital status. Resource utilization outcomes included operative time, number of blood units transfused, length of stay, number of days in intensive care unit (ICU), readmissions, and reoperation.

Standardized mean differences (SMD) were used to assess similarity in baseline demographics between the two groups (barbed TCS vs. conventional PDS suture). Multivariate adjusted analyses were conducted for outcomes with sufficient event rates. For this multivariate adjustment, linear regression and logistic regression were used for continuous and categorical variables, respectively. In addition, outcomes such as readmissions and complications were assessed in time-to-event analyses using a Cox proportional hazards model. Baseline covariates (age, sex, BMI category, any surgeries 5 years prior to index procedure, number of comorbidities, medication use at the time of index procedure, mesh reinforcement use, hernia defect size) were included in the multivariate model as the first step, and further backward selection was applied to retain only those covariates with *P* < 0.10. All statistical tests were two-sided, and significance was defined at the 5% level. Unless otherwise specified, all statistical analyses were conducted using R version 3.3.1 (or later) and SAS version 9.4 TS1M4 (Cary, NC). This research is reported in line with the Strengthening the Reporting of Cohort Studies in Surgery (STROCSS) criteria [7].

## Results

A total of 821 open hernia procedures were performed between January 1, 2013, and August 31, 2020, of which 446 using barbed TCS and 375 using conventional PDS suture (uncoated used initially in the study period, with a switch to triclosan-coated later). At 30-day follow-up, complete records were available for 412 of barbed TCS and 374 PDS suture patients; the 60- and 90-day follow-up analyses included 371 barbed TCS and 369 conventional PDS suture patients. Baseline demographic characteristics were similar between the barbed TCS and conventional PDS groups, except for the 5-year surgical history. The proportion of patients who underwent abdominal surgery or a midline laparotomy in the 5 years prior to the index procedure was significantly higher in the conventional PDS suture group (*P* < 0.0001) (Table 1).

**Table 1:** Baseline demographic and clinical characteristics of patients undergoing open hernia repair by type of suture

	Type of suture		SMD	*P*-value
	Barbed TCS	Conventional PDS suture		
	*n* = 446	*n* = 375		
Age at index				
Median [IQR]	61.8 [52.3; 68.9]	61.3 [51.3; 69.0]	0.0739	0.3995
Age groups at index, *n* (%)				
< 50 years	92 (20.6%)	89 (23.7%)	0.0726	0.5633
50–64 years	181 (40.6%)	147 (39.2%)		
≥ 65 years	173 (38.8%)	139 (37.1%)		
Sex, *n* (%)				
Male	222 (49.8%)	196 (52.3%)	0.0498	0.477
Female	224 (50.2%)	179 (47.7%)		
BMI				
Median [IQR]	27.4 [24.6; 31.2]	27.7 [24.5; 31.6]	0.007	0.8266
BMI categories, *n* (%)				
Underweight (< 18.5 kg/m^2^)	5 (1.1%)	3 (0.8%)	0.1318	0.2263^a^
Normal (18 to < 25 kg/m^2^)	121 (27.1%)	105 (28.0%)		
Overweight (25 to < 30 kg/m^2^)	187 (41.9%)	134 (35.7%)		
Obese (≥ 30 kg/m^2^)	133 (29.8%)	133 (35.5%)		
Any hernia repair 5 years prior to the index procedure, *n* (%)				
Yes	6 (1.3%)	9 (2.4%)	− 0.0779	0.261
No	440 (98.7%)	366 (97.6%)		
Any surgeries 5 years prior to the index procedure, *n* (%)				
Yes	217 (51.3%)	268 (71.5%)	− 0.4234	< 0.0001
No	206 (48.7%)	107 (28.5%)		
Number of comorbidities				
Median [IQR]	1.0 [0.0; 2.0]	1.0 [0.0; 2.0]	0.2349	0.0012
Number of comorbidities, *n (%)*				
0	128 (28.7%)	132 (35.2%)	0.2404	0.0151
1	118 (26.5%)	117 (31.2%)		
2	94 (21.1%)	68 (18.1%)		
3	61 (13.7%)	32 (8.5%)		
4 +	45 (10.1%)	26 (6.9%)		
Medication use at the time of the index procedure,* n (%)*				
Anticoagulation medicine	112 (25.1%)	25 (6.7%)	0.5214	< 0.0001
Cortisone	10 (2.2%)	30 (8.0%)	− 0.2635	0.0001
Immunosuppressive drugs	37 (8.3%)	28 (7.5%)	0.0308	0.6611
Use of mesh reinforcement, *n (%)*				
Yes	403 (90.4%)	355 (94.7%)	− 0.16423	0.0209
Mesh repair method, *n* (%)	*n* = 403^b^	*n* = 355^b^		
Onlay	17 (4.2%)	4 (1.1%)	0.22042	0.0115
Sublay	363 (90.1%)	320 (90.1%)		
IPOM	23 (5.7%)	31 (8.7%)		
Hernia defect size (cm^2^)				
Median [IQR]	36.0 [15.0; 100.0]	46.5 [16.0; 108.0]	− 0.0865	0.0378

*SMD* standardized mean difference, *SD* standard deviation, *IQR* interquartile range, *BMI* body mass index

^a^ Fisher’s exact test was used instead of Chi-square test

﻿^b^ Denominator is the number of patients that used mesh reinforcement

Significant differences were observed between the two groups in the average number of comorbidities (mean [SD] 1.5 [1.4] barbed TCS vs. 1.2 [1.3] conventional PDS; *P* = 0.0008). The comorbidity burden was higher in barbed TCS than in conventional PDS patients (23.8% vs. 15.4% for ≥ 3 comorbidities). The barbed TCS group included significantly more patients with myocardial infarction/congestive heart failure (21.3% vs. 14.9%, *P* = 0.019), rheumatic disease (2.0% vs. 0.0%, *P* = 0.0048) and arterial hypertension (28.5% vs. 19.2%, *P* = 0.002) than the PDS group. PDS patients were more likely to have moderate to severe liver disease than barbed TCS patients (9.3% vs. 5.8%, *P* = 0.0113). Significantly greater numbers of barbed TCS patients were using anticoagulants (25.1% vs. 6.7%; *P* < 0.0001), while a greater number of conventional PDS patients were using cortisone products (8.0% vs. 2.2%; *P* = 0.0001) (Table 1).

Mesh reinforcement was used in significantly fewer barbed TCS patients than conventional PDS patients (90.4% vs. 94.7%; *P* = 0.0209) (Table 1). The distribution by mesh repair method was different between the two cohorts, with onlay greater in barbed TCS patients (4.2% vs. 1.1%) and intraperitoneal onlay mesh (IPOM) higher in conventional PDS patients (8.7% vs. 5.7%). Likewise, the distribution by mesh fixation method was different between groups with suture used more often in barbed TCS patients (93.5% vs. 76.3%) and self-gripping more often in conventional PDS patients (21.4% vs. 5.5%). All patients that received a mesh had a standard dosage of antibiotics once, type cephalosporin grade 2.

### Clinical outcomes

**Fig. 1:**
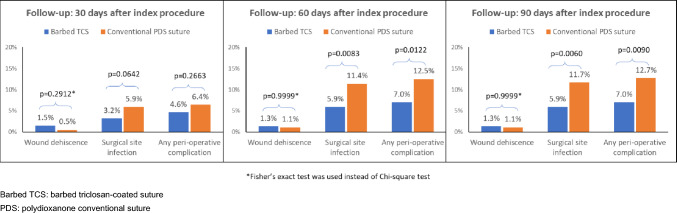
Clinical outcome assessment during follow-up

At 30-day follow-up, clinical outcomes were not significantly different between the barbed TCS and conventional PDS groups for wound dehiscence, SSIs, and perioperative complications (Fig. 1). However, the relative frequency of SSIs at the 60- and 90-day follow-up was significantly greater in the conventional PDS group (60 days, 11.4% vs. 5.9%; *P* = 0.0083; 90 days 11.7% vs. 5.9%; *P* = 0.0060). Overall perioperative complications occurred more frequently in conventional PDS patients both at 60-day (12.5% vs. 7.0%; *P* = 0.0122) and 90-day follow-up (12.7% vs. 7.0%; *P* = 0.0090) (Fig. 1). Among patients developing SSIs, deep SSIs at 60- and 90-day follow-up were also significantly more frequent in the conventional PDS group (60 days, 15/42 vs. 2/22, 35.7% vs. 9.1%; *P* = 0.022; 90 days, 15/43 vs. 2/22, 34.9% vs. 9.1%; *P* = 0.0252). Despite these results, the need for surgical treatment of a site infection was not different between groups. Likewise, there was no significant difference in the number of patients who received blood transfusion or in the number of deaths at any follow-up assessment.

Multivariate analyses showed that at 30, 60, and 90 days, there was no significant association between the type of suture used and wound dehiscence outcome when adjusted for confounders (Table 2). At 30-, 60- and 90-day follow-up, barbed TCS patients were nearly half as likely to experience SSIs (Table 2). The likelihood of any perioperative complication was non-significant for suture type at 30 days, but at 60- and 90-day follow-up, barbed TCS patients were nearly half as likely to experience perioperative complications as compared to conventional PDS suture (Table 2). No differences were seen for the different categories of intraoperative complications, such as any intraoperative complication, bladder injury, bowel lesion, severe bleeding, general complication, or other intraoperative complications.

**Table 2:** Multivariate model-adjusted clinical outcomes for patients undergoing open hernia repair using barbed TCS vs. conventional PDS suture

	Type of suture	*P* value
	Barbed TCS vs. conventional PDS suture (ref)	
Follow-up: 30 days after the index procedure		
Wound dehiscence, OR [95% CI]	2.43 [0.47–12.59]	0.2912
Surgical site infection, OR [95% CI]	0.47 [0.22–0.98]	0.0455
Any perioperative complication, OR [95% CI]	0.68 [0.36–1.30]	0.2419
Follow-up: 60 days after the index procedure		
Wound dehiscence, OR [95% CI]	1.04 [0.26–4.18]	0.9583
Surgical site infection, OR [95% CI]	0.49 [0.28–0.88]	0.0158
Any perioperative complication, OR [95% CI]	0.53 [0.30–0.91]	0.0204
Follow-up: 90 days after the index procedure		
Wound dehiscence, OR [95% CI]	1.04 [0.26–4.18]	0.9583
Surgical site infection, OR [95% CI]	0.48 [0.27–0.85]	0.0122
Any perioperative complication, OR [95% CI]	0.48 [0.28–0.81]	0.0067

There were more intrahospital hematoma complications in the barbed TCS cohort than in the conventional PDS cohort (3.8% vs. 1.3%; *P* = 0.0285). Intrahospital cardiac complications also occurred more frequently in barbed TCS patients (2.5% vs. 0.5%; *P* = 0.0271). No differences in relative frequency were observed for other intrahospital complications (e.g., bleeding, respiratory complications, renal complications, nerve damage, or other general complications).

### Resource utilization

There was no difference between the two suture type groups in terms of blood units transfused. Virtually, no ICU time was required by patients in either group.

The three resource outcomes that differed between the two patient groups were operative time, days of surgical drainage overall, and hospital LOS. On average, mean time in surgery was about 25 min longer for barbed TCS patients (mean [SD]: 170.4 [102.1] vs. 145.3 [70.5] min; *P* < 0.0001). Time in surgery ranged from 12 to 600 min across the cohorts. The mean duration of surgical drainage was statistically longer in conventional PDS patients (mean [SD]: 3.3 [1.1] days vs. 3.1 [1.4]; *P* = 0.0053), where the absolute difference of 0.2 days was equal to 4.8 hours. Hospital LOS was also longer in the conventional PDS group by an average of 1.8 days (mean [SD]: 8.3 [12.2] days vs. 6.5 [6.0]; *P* = 0.013).

Throughout the follow-up periods, there was no apparent difference in the number of reoperations, number of days between the index procedure and reoperation, distribution of reasons for reoperation, presence of wound debridement during reoperation, use of negative pressure therapy, or need for re-exploration. Furthermore, there were no significant differences in the cumulative number of readmissions, readmission LOS, or ICU use between barbed TCS and conventional PDS patients.

Multivariate analyses revealed significant differences between suture groups in the adjusted mean operative time, mean hospital LOS, and mean number of surgical drainage days. Barbed TCS patients took an average 27 min longer in surgery than conventional PDS patients (Table 3). However, PDS patients stayed in the hospital for an average of 2.48 days longer than barbed TCS patients. Conventional PDS patients also had longer average time of surgical drainage by 0.28 days or 6.7 hours (Table 3). At 30-, 60-, and 90-day follow-up, no association between type of suture and readmission, or reoperation was observed (Table 3).

**Table 3:** Multivariate model-adjusted resource utilization outcomes for patients undergoing open hernia repair using barbed TCS vs. conventional PDS suture

	Type of suture	
	Barbed TCS	Conventional PDS suture
	*n* = 446	*n* = 375
Index procedure		
Operative time, mean [95% CI]	171.29 [163.28–179.31]	144.31 [135.56–153.05]
Hospital LOS, mean [95% CI]	5.70 [4.92–6.62]	8.18 [7.33–9.14]
Number of surgical drainage days, mean [95% CI]	3.08 [2.96–3.20]	3.36 [3.23–3.49]
Follow-up: 30 days after the index procedure	Barbed TCS vs. conventional PDS suture (ref)	
Readmission after the index procedure hospitalization, OR [95%CI]	4.87 [0.57–41.85]	
Reoperation, OR [95%CI]	1.42 [0.37–5.39]	
Follow-up: 60 days after the index procedure	Barbed TCS vs. conventional PDS suture (ref)	
Readmission after the index procedure hospitalization, OR [95%CI]	5.25 [0.61–45.15]	
Reoperation, OR [95%CI]	2.73 [0.81–9.18]	

### Time-dependent analyses

**Fig. 2:**
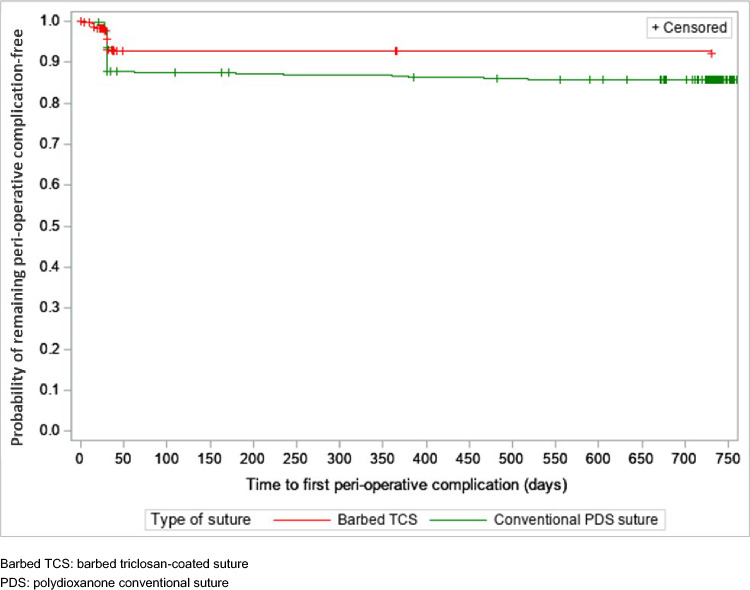
Kaplan–Meier curve for time to any perioperative complication for wound dehiscence, surgical site infection, hernia recurrence, and other complications

The use of a particular suture was not associated with the risk of reoperation (HR [95%CI] for barbed TCS vs. conventional PDS: 1.27 [0.48–3.39]; *P* = 0.4793). Use of barbed TCS was associated with a significantly reduced risk of perioperative complications over the follow-up period as compared to conventional PDS (HR [95% CI]: 0.52 [0.33–0.83]; *P* = 0.0058). Figure 2 shows the risk reduction in perioperative complications after hernia repair. The presence of comorbidities increased the risk of perioperative complications by 22% with the presence of each selected comorbid condition (HR [95% CI]: 1.25 [1.08–1.46]; *P* = 0.0037).

## Discussion

In our single-center retrospective cohort study, no significant differences were observed between barbed TCS and conventional PDS suture for outcomes of wound dehiscence, reoperation, and readmission. At 30-day follow-up, the results indicated that patients in both groups recovered equally well. While there were less perioperative complications in the barbed TCS patients, the observed differences were not significant. Some significant differences were observed at the 60- and 90-day follow-up, in both univariate and multivariate analyses. Barbed TCS patients experienced significantly less SSI and perioperative complications than conventional PDS suture patients. Furthermore, among patients who developed SSI, deep site infections were also significantly less frequent in barbed TCS patients at 60 and 90 days. It was hypothesized that the mixed use of triclosan-coated and uncoated suture in the conventional PDS suture group might be the cause of the observed higher rates of SSI, driven by the use of uncoated suture material in a subgroup of patients. Further in terms of resource utilization, operative time was statistically significantly longer, while the number of surgical drainage days and overall LOS were statistically significantly lower for barbed TCS vs. conventional PDS suture.

The development of barbed suture materials was an important evolutionary step in wound closure surgery, offering a safe and effective alternative to conventional suture materials [8–11]. The main advantages consist in a faster closure time [8–11], by eliminating the need for knot tying and assistance for suture placement, and an even distribution of tension, which make such materials a better alternative for surgical closure of fascia and other high-tension wounds [7, 8]. There is a lack of consensus regarding the best surgical approach to use in abdominal wall closure to prevent complications [2]. Given the vast choice of suturing materials available on the market, evidence is based on studies with relatively small sample sizes and variable postoperative follow-up. A recent network meta-analysis of 31 trials, including data on 11,533 patients undergoing abdominal wall repair, used pairwise comparisons of suture materials for assessment of clinical outcomes [4]. The results revealed that no suture material outperformed the rest in terms of prevention of SSI, midline incisional hernia, wound dehiscence, or sinus/fistula occurrence [4].

Previous studies have shown that TCS can reduce SSI risk [12]. The results of a systematic review and meta-analysis of 25 RCTs with a pooled population size of 11,957 concluded that TCSs significantly reduced the risk of SSI at 30 days postoperatively [13]. Similarly, a systematic review and meta-analysis, focused on the efficacy of fascial closure using antimicrobial sutures specifically for the prevention of SSIs in gastrointestinal surgery, confirmed that antimicrobial-coated sutures significantly lowered the risk of incisional SSIs compared with non-coated sutures [6]. As SSIs can manifest post the 30-day mark, capturing SSIs at a 90-day follow-up was shown to be beneficial in several studies including in open hernia repair [14, 15]. We believe that eliminating the need for surgical knots, a potential nidus for infections, could result in less frequent wound infections when using barbed suture [16]. Another point of entry for bacteria are surgical drains, and several studies have shown their association with higher wound infection rates in different types of surgical interventions [17–19]. Further, the higher prevalence of moderate to severe liver disease and higher use of cortisone products in conventional PDS patients as compared to barbed TCS patients observed in this study may be tied to an elevated risk of infection. While the need for surgical treatment of a site infection was no different between the groups, the significantly longer LOS in the conventional PDS group may reflect, at least in part, the observed higher SSI risk. Deep SSIs were found to be a predictor of increased LOS, readmission risk, and additional costs [20].

In the present study, even though surgical drainage was significantly shorter in barbed TCS patients by 0.28 days, the equivalent of 6.7 hours, we believe this difference was not clinically important. Similarly, a retrospective analysis of 117 abdominal wall reconstruction surgeries that examined the role of varying surgical drainage duration periods on the occurrence of complications showed that the duration of drainage was not a predictor of wound complications [21]. Furthermore, in our study, the need for surgical treatment of a site infection or for blood transfusion was not different between the groups, and no differences were observed in the number of deaths at any follow-up assessment point.

Elective ventral hernia repairs can be associated with increased utilization of healthcare resources, mainly related to prolonged hospital stay and readmissions due to pain and surgical complications [22]. In the present study, although observed differences in LOS between suture groups were significant, resource utilization overall was mostly similar. Although barbed TCS procedures took longer in surgery than conventional PDS, the difference in total operative time was more likely due to the hernia procedure itself. The broad range for operative time was driven by the type of hernia procedure, with brief operative time for trocar hernias and extensive procedures for giant midline incisional hernias with loss of domain.

There were no significant differences observed for blood units transfused, ICU use, number of and reasons for reoperations, number of days between index procedure and reoperation, presence of wound debridement during reoperation, use of negative pressure therapy, or need for re-exploration. Furthermore, there were no significant differences in cumulative number of readmissions, readmission LOS or ICU use between barbed TCS and conventional PDS patients.

No difference was observed for intraoperative complications. However, intrahospital hematoma and cardiac complications appeared more frequently in barbed TCS users, which may be due to a higher baseline prevalence of myocardial infarction and use of anticoagulants in this group. The overall numbers of these complications were low, and the severity of the events is not known. It is unlikely that the sutures had any role in these events. Survival analyses showed no risk difference over time between type of suture and reoperation. However, use of barbed TCS significantly reduced the risk of perioperative complications over time following the procedure, beginning in the 30 to 60-day period after hernia repair.

There are certain limitations to this study. The use of a retrospective design is limited by the accuracy and completeness of recorded data and opens the possibility for confounding due to unmeasured covariates. This study used data from a single center, thus ensuring consistency in recording variables of interest, and only patients with complete records were considered for inclusion in the study. Although most single-center studies have relatively small study populations and no formal sample size calculations were performed for this study, our large practice offered the possibility of having the sample size required to detect significant differences between the two groups of interest. By expanding the follow-up to 90 days post-surgery, we increased the likelihood of capturing short-term complications, as more than 90% of SSIs and 80% of readmissions after open hernia repairs are known to occur within 90 days [14]. Another limitation is the calendar time difference between the use of the two types of sutures, with conventional PDS sutures used in an earlier period than the barbed sutures, and the potential that there may have been improvements in SSI and hernia complication rates during this time. However, the differences between the conventional PDS and barbed suture periods were very large, with reductions of 50–60% during the two time periods that were unlikely to be accounted for by secular trends. In addition, a targeted literature review did not identify any discernible trends in SSI or hernia complication reduction over the study period.

## Conclusions

This single-center retrospective cohort study shows that patients who underwent open hernia repair appeared to recover equally well regardless of the suture type. Survival analyses showed no risk difference over time between the type of suture and reoperation. However, use of STRATAFIX™ Symmetric PDS Plus suture was associated with a statistically significant reduction in the risk of perioperative complications over time as compared to conventional PDS suture, beginning in the 30- to 60-day period after hernia repair.

### Supplementary Information

Below is the link to the electronic supplementary material.

Supplementary file1 (PDF 76 KB)

## Data Availability

Not applicable.
