# Supplementary material for: Clinical outcomes of triclosan-coated barbed suture in open hernia repair: a retrospective cohort study
Source: Hernia. 2024 Apr 12;28(4):1239–47. doi: 10.1007/s10029-024-03012-0 (PMC11297053; doi:10.1007/s10029-024-03012-0)
Supplement: Supplementary file 1 — Supplementary file1 (PDF 76 KB) [file 10029_2024_3012_MOESM1_ESM.pdf]

Operative Technique

The procedure as used in this series starts with a midline incision that is carried down through the subcutaneous tissues until the anterior fascia is reached. The hernia sac is then identified and the fascia superior or inferior to the defect is entered. This fascial incision is lengthened to ensure that there is adequate exposure of the hernia defect. After careful dissection the peritoneum is always opened carefully with full exploration and adhesiolysis of the abdomen.

After the intraperitoneal portion of the procedure is complete, the retromuscular plane can be created for mesh placement. Observing the posterior rectus sheath from underneath, this layer is incised and dissected away from the rectus abdominis. Caution and use of blunt dissection during this portion of the case ensures preservation of the segmental neurovascular bundles and inferior epigastric vessels. If necessary, the fatty triangle behind the xiphoid process is opened and freed over 4-5cm to increase later mesh overlap. If the hernia defect extends below the arcuate line, the transversalis fascia with peritoneum is dissected away from the anterior structures and carried down even more caudally into the spaces of Retzius and Bogros, exposing the pubic symphysis.

Laterally, the dissection typically extends to the lateral border of the rectus sheath in a typical repair according to Rives and Stoppa, but if necessary to extend mesh overlap a Transverse Abdominis Release (TAR) is carried out on one or both sides across the linea semilunaris.

When full mobilization is achieved posteriorly, the posterior layer is then closed using a resorbable Vicryl 2/0 suture, and mesh (large pore monofilament polypropylene or polyvinylidene fluoride (PVDF)) is placed to reinforce the repair. Once the mesh is in place with an overlap of at least 5cm to all sides, it is fixed to bony edges using a multifilament suture (Ti-cron 1) or to the posterior layer using separate resorbable sutures (Vicryl 3/0) for each quadrant. Two closed suction drains are positioned above the mesh through lateral stab wounds and removed at postoperative day 2.

Next, the anterior fascia is sutured together and the midline is completely reconstructed using mesh augmentation. If there is persistent tension on the midline after retromuscular placement of mesh, an anterior component separation according to Ramirez is performed on one or both sides, but only when a TAR was not performed at that same side earlier. This maneuver allows for added laxity of the midline, leading to less strain on the fascia when brought back together. The anterior fascia is then sutured using PDS 1 or a barbed triclosan coated suture 1 or 0 (Stratafix Symmetric PDS Plus 1 or 0).

In case a bilateral anterior component separation technique was needed, in several cases an intraperitoneal mesh was placed to achieve wide mesh overlap covering both semilunar lines till the

mesh reaches the psoas muscle. In these cases a running PDS 2/0 was used all around to fix the mesh to the peritoneum.

In all cases 1 or 2 suction drains are placed subcutaneously to prevent seroma formation and these are removed when daily output is less than 30cc/day.

An abdominal binder was used in all patients for 3 weeks to increase mobilization and patient comfort during activities.
